# Synergistic Effects of Clopidogrel and Fufang Danshen Dripping Pills by Modulation of the Metabolism Target and Pharmacokinetics

**DOI:** 10.1155/2014/789142

**Published:** 2014-11-02

**Authors:** Shitang Ma, Wenzheng Ju, Guoliang Dai, Wenzhu Zhao, Xiaogui Cheng, Zhuyuan Fang, Hengshan Tan, Xiaoxiao Wang

**Affiliations:** ^1^College of Pharmacy, Nanjing University of Traditional Chinese Medicine, Nanjing, Jiangsu, China; ^2^Food and Drug College, Anhui Science and Technology University, Fengyang, Anhui, China; ^3^Department of Clinical Pharmacology, Affiliated Hospital of Nanjing University of Traditional Chinese Medicine, No. 155 Hanzhong Road, Baixia District, Nanjing, Jiangsu 210029, China; ^4^Department of Cardiology, Affiliated Hospital of Nanjing University of Traditional Chinese Medicine, Nanjing, China; ^5^Department of Clinical Pharmacology, General Hospital of Nanjing Military Area Command, Nanjing, China

## Abstract

*Background and Objective.* The aim was to evaluate the synergistic effects of clopidogrel and FDDP by modulating the metabolism target and the pharmacokinetics. *Methods*. The inhibition effect of FDDP on the CES1 was first investigated by the molecular simulation method, and the synergistic effects on the pharmacokinetics of CPGS were studied as follows: SD rats were treated with oral clopidogrel alone at a dosage of 30 mg/kg or the combination of clopidogrel and FDDP at dosages of 30 mg/kg and 324 mg/kg, respectively, for 21 days. The concentrations of CPGS in the blood plasma samples were determined and the calculated concentrations were used to determine the pharmacokinetic parameters. *Results*. 20 compounds in FDDP potentially interacted with CES1 target. The CPGS showed a two-compartment model pharmacokinetic profile. The concentration-time course of CPGS was not changed by FDDP, but FDDP decreased the peak plasma concentration and area under the curve of CPGS. *Conclusion*. The CES1's activity could be partly inhibited by FDDP through the molecular simulation investigation. The concentration-time course of CPGS was altered slightly by FDDP. The results demonstrated the synergistic effects of clopidogrel and FDDP by modulating both the pharmacokinetics and the target metabolism.

## 1. Introduction

Antiplatelet therapy with clopidogrel (an ADP P2Y12 receptor antagonist) is recommended by current clinical practice guidelines to prevent atherothrombotic event in patients with coronary artery disease (CAD) [1]. Despite the obvious advantages of the clopidogrel, many clinical studies have shown that roughly 5–40% of patients treated with conventional doses of clopidogrel display inadequate antiplatelet responses [2], which may lead to serious clinical consequences.

Clopidogrel is a prodrug and requires complex metabolic activation. In the liver, 85% of the absorbed parent drug can be transformed by carboxyl esterase 1 (CES1) into an inactive carboxylic acid derivative of clopidogrel (CPGS) [3], the most abundant species circulating clopidogrel in blood. On the other hand, only 15% of the absorbed clopidogrel dose is transformed by cytochrome P450 (CYP) into a thiol metabolite, which is afforded the antiplatelet effect of clopidogrel [4].

The partial inhibition of CES1 activity would lead to the decline of CPGS production and the increase of active thiol metabolite. Some research has demonstrated that the high concentration of CPGS accumulation is related to clopidogrel resistance, as the plasma concentration of the parent clopidogrel goes down very fast and the active metabolite from the CYP450 pathway is highly labile [5]. Since neither the parent nor the active thiol metabolite of clopidogrel is detected in plasma due to low concentration [6], the quantization of inactive CPGS is believed to be a better measure as an indirect approach for studying* in vivo* measurement of pharmacodynamic effect by estimating platelet aggregation.

The Chinese traditional medicine for treating CAD, especially for activating blood circulation to promote blood stasis, is commonly used [7, 8]. Fufang Danshen Dripping Pill (FDDP, a patent medicine approved by SFDA with Chinese drug number Z10950111) is a famous traditional Chinese medicine (TCM) recipe containing* Salvia miltiorrhiza* (SM), Radix notoginseng (NG), and Borneol (BN) and has been used for the treatment of CAD [9]. Many of the prescriptions from the TCM hospital for the treatment of some CAD usually contain FDDP and clopidogrel [10]. To our knowledge, there has not yet been a publication describing a synergistic effect of clopidogrel and FDDP by modulating the metabolism target. And there is no research regarding the interplay of the combination on the pharmacokinetics* in vivo* at present; it is therefore necessary to investigate the rationality of combined applications and interaction of the two drugs on the metabolism target and pharmacokinetics.

## 2. Experimental

### 2.1. Reagents and Materials

CPGS (lot number 2-pll-60-3) and caffeic acid (IS, lot number 110885-200102) were purchased from the Toronto Research Chemicals Inc. and National Institute for the Control of Pharmaceutical and Biological Products. FDDP, 10 mg/pill, lot number 130210, was manufactured by Tianjin Tasly Pharmaceutical Co. (Tianjin, China). Clopidogrel tablets, 25 mg/tablet, lot number AA20130207, expiring before January 2016, were manufactured by Shenzhen Salubris Pharmaceuticals Co. (Shenzhen, China). Acetonitrile and methanol were of HPLC grade (Merck, USA). All other chemicals used were of AR grade. Ultrapure water used for the HPLC was from the Milli-Q water purification system (Millipore, USA).

### 2.2. Preparation of the FDDP Bioactive Components

One hundred twenty-seven bioactive compounds were first identified in the three herb medicines SM, NG, and BN through pharmacochemistry and PK by HPLC-MS [11–13] methods and database searches using the Phytochemical and Ethnobotanical Database of the Chinese Academy of Sciences and Dr. Duke. Maestro was employed to set up the database.

The key descriptors including AlogP, Estate, HBA, HBD, MW, MR, PHOA, polarity, PSA, RB, and ROF were calculated using the Maestro Canvas module. These key parameters can reflect the basic characteristics of drug- (lead-) like and PK-PD properties [14]. The max, mean, median, min, mode, variance, and standard deviation values of these key descriptors were calculated to describe the basic characteristics of FDDP.

#### 2.2.1. Preparation of the CES1 Target

The CES1 target protein was retrieved from the RCSB Protein Data Bank Database (PDB ID 1MX1) [15]. The Maestro protein preparation wizard was employed as below: the water molecules, the geometry of all of the hetero groups, and the hydrogen atoms were first processed. Second, the prime module tool was selected to predict to fill in missing loop side-chains. The position of the hydrogen bonds was finally optimised using the constraint of the OPLS_2005 force field. After preparation, the binding site grids were generated by protein grid generation module.

#### 2.2.2. The Molecular Docking Study between the Components and the CES1 Target

To elucidate the biological affection between the FDDP components and the CES1 target, a molecular docking simulation was performed using Maestro Glide (version 5.0). The prepared one hundred twenty-seven molecules and the CES1 target were subjected to a Glide based three-tiered in silico target screening strategy through two stages: the first stage of HTVS docking screened the compounds that were retrieved, and all screened compounds underwent the second stage, a more computationally intensive and accurate SP mode screening. The docking score, constituted with binding affinity, hydrogen-bonding, and glide energy, was eventually calculated. The original ligand, tacrine, bound to the CES1 receptor was also docked into the respective grid binding site, using the docking score of the protein-tacrine complex as the cutoff.

### 2.3. Preparation of Standard Solutions and Plasma Samples

The standard stock solutions were prepared by dissolving CPGS (1000 *μ*g/mL) in acetonitrile. Working solutions were prepared from the stock solutions by dilution in acetonitrile. All working solutions were stored at 4°C. A solution of caffeic acid (IS) was diluted to a final concentration of 1.20 *μ*g/mL using acetonitrile. The calibration standards and QC samples were prepared by spiking blank plasma with the combined working solutions. The standard concentrations were 0.3, 0.6, 1.2, 2.5, 5.0, 10.0, and 20.0 *μ*g*·*mL^−1^. The standard calibration samples and QC samples were stored at −20°C.

### 2.4. HPLC Instrumentation and Conditions

Analyses were performed using the Agilent HPLC 1100 system (Agilent, USA) consisting of a quat pump (G1311A), an autosampler (G1313A), and an online degasser (G1322A). The chromatographic separation was performed on an Agilent Zorbax SB C18 column (4.6 mm × 250 mm, 5 *μ*m). A simple gradient method was used, in which the percentage of acetonitrile-buffer (0.2% phosphoric acid in water) was varied as shown in Table 1 at a flow rate of 1.0 mL/min. The injection volume was 10 *μ*L. The total LC run time was 12 min with the column temperature kept at 30°C and the detection at a wavelength of 220 nm.

### 2.5. Procedure for Sample Extraction

An aliquot of 10 *μ*L 1 M hydrochloric acid (HCL), 100 *μ*L blood plasma, and 10 *μ*L of IS (160 *μ*g/mL) was added into a 1.5 mL centrifuge tube. After vortex-mixing for 1 min, 1 mL of extraction solvent (ethyl acetate-dichloromethane; 4 : 1, V/V) was added and vortexed vigorously for 4 min, followed by centrifugation for 12 min at 12000 rpm. Next, 950 *μ*L of supernatant was transferred into another tube and evaporated to dryness at 40°C for 90 min under a gentle stream of nitrogen. The residue was reconstituted with 100 *μ*L of the mobile phase and vortexed for 1 min and then centrifuged for 5 min at 12000 rpm. For analysis, 10 *μ*L of the supernatant was injected onto the HPLC system. The plasma concentrations of the analytes exceeded the upper limit of quantification, and therefore the sample was diluted with blank plasma and vortexed for 1 min. A 100 *μ*L aliquot of diluted sample was processed under the same sample pretreatment procedure as mentioned above.

### 2.6. Method Validation

The method was validated for specificity, sensitivity, linearity, precision and accuracy, recovery, and stability [16].

The specificity was evaluated by comparing the chromatography of six randomly selected rats. The drug-free plasma samples were processed and injected into HPLC to assess the extent to which endogenous plasma components may interfere with the retention times of the analyte and IS.

The lowest limit of quantification (LLOQ) blood sample should be discrete, identifiable, and reproducible in five replicates with the precision of ±20% using the relative standard deviation (RSD) and accuracy within 80–120% of the spiked concentration. The deviation of standards other than LLOQ should be no more than ±15% of the nominal concentration. It was desirable that a minimum of five nonzero standards, including LLOQ, met the above criteria.

The linearity for each plasma analyte was evaluated by analysing the calibration curves from seven nonzero concentrations of calibration samples in duplicate in five separate runs. Blank plasma samples were analysed to discard the presence of interference. The calibration curves were plotted by a weighted least squares linear regression method (weighting factor = 1/*x* ^2^) through the measurement of the peak area ratio of the CPGS to IS.

Recoveries of CPGS were determined at three different concentration levels by comparing the peak areas of extracted samples (spiked before extraction) with those from the standard solutions at the same concentrations.

The intrabatch precision and accuracy were evaluated in five replicate analyses for CPGS at three QC levels on the same analytical run. Interbatch precision and accuracy were calculated after repeated analysis in three different analytical runs. Concentrations were calculated from the calibration curve. The accuracy and precision were calculated and expressed in terms of % bias and relative standard deviation (%RSD), respectively.

Stability experiments were performed to evaluate the analyte stability in stock solutions and in plasma samples under different conditions. Room temperature stability, refrigerated stability of extracted sample, freeze-thaw stability, and long-term stability were performed using three replicates at each QC level.

### 2.7. Pharmacokinetic Study

The preclinical pharmacokinetic study was based on 16 SD rats. The investigation was approved by the Animal Ethics Committee of the Nanjing University of Traditional Chinese Medicine. Two groups of 8 rats each were used according to a parallel study. The clopidogrel-only group received 30 mg/kg oral administration of clopidogrel. The combination group (clopidogrel 30 mg/kg + FDDP 324 mg/kg) received their dose administered as a combination. The rats fasted for 12 h prior to the experiment, while free access to water remained. Plasma samples were collected at 0, 0.25, 0.5, 1, 1.5, 2.3, 4, 6, 8, 10, 12, and 24 h after dosing. Blood samples of 400 *μ*L were obtained via an in-dwelling catheter in the caudal vein and collected into heparinized tubes. Then, the plasma was centrifuged for 10 min at 4000 rpm and the supernatant was transferred to labelled plastic vials at −80°C until the completion of the analysis.

### 2.8. Pharmacokinetic Analysis

Pharmacokinetic parameters were calculated using Drug and Statistic (DAS) 3.0 pharmacokinetic software (Chinese Pharmacological Association, Anhui, China). The pharmacokinetic parameters included the compartment parameters and statistical parameters (Table 6).

The area under the plasma concentration-time curve (AUC) was calculated using the linear trapezoidal rule. The elimination rate constant (kel) was calculated by linear regression of the terminal points of the semilog plot of the plasma concentration against time. The half-life of drug elimination during the terminal phase (*t*_1/2_) was calculated from the formula *t*_1/2_ = 0.693/kel. Mean residence time (MRT) was calculated as AUMC/AUC, where AUMC is the area under the first moment of the plasma concentration-time curve. The volume of distribution (Vd) of the central compartment was calculated as dose/*C*_0_, where *C*_0_ is the concentration measured just after the administration. Plasma clearance (CL) was calculated as dose/AUC.

The data are presented as the mean ± SD. Comparisons of the pharmacokinetic data were performed by variance (Student's *t*-test) and the statistically significant difference was set at a value of *P* < 0.05 (SPSS statistical software package, Version 17.0, SPSS Inc., Chicago, IL, USA).

## 3. Results and Discussion

### 3.1. Chemical Distribution of the Bioactive Ingredients Database

One hundred twenty-seven bioactive components were identified in FDDP, and some key molecular descriptors associated with drug- (lead-) like and PK-PD properties are statistically analysed in Table 2. Most of the compounds did not violate Lipinski's rule of five (ROF); the modes of the number of hydrogen bond acceptors (HBA), hydrogen bond donors (HBD), atomic logP (AlogP), and polar surface area (PSA) were 4.08, 2.03, 3.70, and 73.96, respectively. Additionally, the min, mean, median, mode, and standard deviation of the violations of the rule of five (RF) were 0, 0.74, 0, 0, and 0.940, respectively (less than 1). These data suggest that the bioactive database contained drug- (lead-) like and PK-PD-like constituents.

### 3.2. The Molecular Docking Result

**Figure 1:**
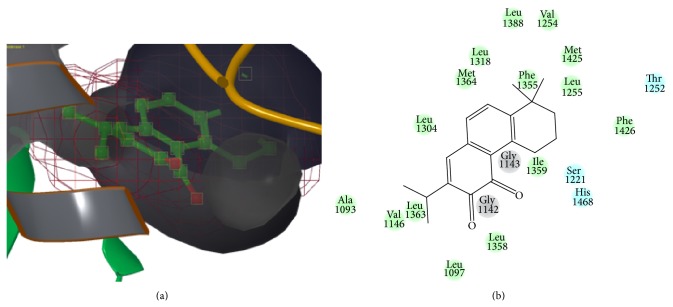
The binding site and mode of miltirone-CES1 complex. (a) The binding site, (b) key interaction residue between compound miltirone and the target human liver carboxylesterase 1 (PDB ID 1MX1).

The bioactive components were contained in FDDP which included two types: water soluble (such as salvianolic acid and saponins) and liposoluble constituent (such as tanshinones). One hundred twenty-seven components were totally identified. HTVS screened compounds from the constructed database were subjected to SP docking based on the docking score, constituted with binding affinity, hydrogen-bonding, and glide energy calculation. 20 compounds (which were shortlisted in Table 3) had positive interaction with the CES1 receptor. Also, the 20 compounds' structures were mainly tanshinone and salvianolic acid and its analogues. Hatfield's literature suggested that the CES's activity could be inhibited by 6 tanshinone analogues through* in vitro* test [17]. Some of our results are consistent with the literature. Beside tanshinone analogues in Hatfield's literature, we also identified that compounds tanshinone IIB, delta-1-dehydrotanshinone, cryptoacetalide, and so forth were also CES1 inhibitor. Since the biological data of miltirone has been reported, the compound was selected to demonstrate the binding mode in Figure 1. Our results indicated that multiple CES1 inhibitors contained in FDDP and the activity of CES1 may be partially inhibited by FDDP through molecular simulation investigations, which may result in the decline of the CPGS concentrations of the CES1 induction pathway.

#### 3.2.1. Selectivity and Sensitivity (LLOQ)

**Figure 2:**
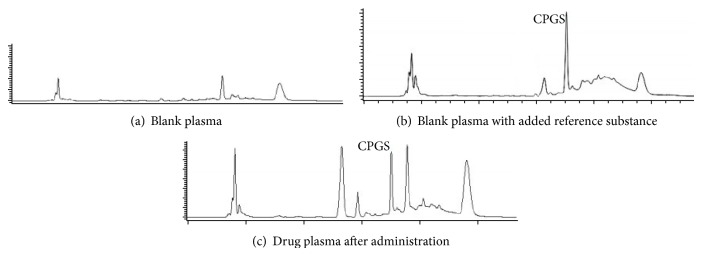
HPLC chromatograms of CPGS in biological CPG samples. (a) Blank plasma; (b) blank plasma with added reference substance; (c) drug plasma after administration. CPGS: carboxylic acid metabolite of clopidogrel.

The liquid-liquid extraction methodology in combination with HPLC detection gave very good selectivity for the analytes and IS.  Figure 2 shows the HPLC chromatography of the spiked plasma with CPGS and IS. The retention time was 6.99 min for CPGS and was 5.23 min for IS. No endogenous interference was detected in the blank plasma samples at the retention time of the analyte and IS. In this case, the LLOQ of CPGS was 0.31 *μ*g/mL.

#### 3.2.2. Linearity and Recovery

For CPGS, the linear regression equation for the mean of five calibration curves was *y* = 16.771*x* + 22.9, *r* ^2^ = 0.9992. The recoveries of CPGS are shown in Table 4. The recoveries of the analyte and IS could be promoted by acidifying the plasma samples with hydrochloric acid; therefore 1 M hydrochloric acid 10 *μ*L was added to the plasma samples before extraction. The recoveries using different organic solvents as extractants were compared. Because the polarities of the analyte varied, ethyl acetate-dichloromethane was chosen as the extractant to obtain suitable recoveries of each analyte.

#### 3.2.3. Accuracy and Precision

The obtained accuracy (between 85% and 115%) and precision (less than 15%) were acceptable. Intrabatch and interbatch precision and accuracy for the analysis of CPGS in rat plasma are presented in Table 5. These data show that the HPLC assay is excellent for the quantitative analysis of analyte in rat plasma.

#### 3.2.4. Stability

CPGS were found to be stable in extracted plasma samples for 24 h at room temperature. Both analytes were stable throughout three freeze/thaw cycles. The CPGS spiked plasma samples stored at −20°C for long-term stability were determined to be stable for 20 days.

#### 3.2.5. Application

**Figure 3:**
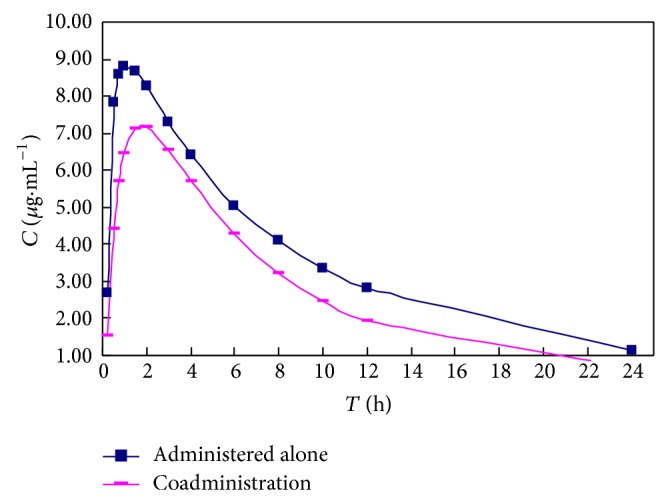
The mean plasma concentration-time profiles of CPGS after intragastric administration to rats of clopidogrel alone or coadministered with Fufang Danshen at doses of 324 mg/kg and 30 mg/kg, respectively, for 21 days.

The curves of the plasma concentration-time courses of the CPGS for clopidogrel administration alone or coadministeration with FDDP are shown in Figure 3. The pharmacokinetics of CPGS could be described by a two-compartment open model. The pharmacokinetic parameters of CPGS are summarised in Table 4. There was no statistically significant effect of clopidogrel on the CPGS concentration-time profiles, unlike the results following the coadministration of clopidogrel with FDDP. The MRT (0-∞) and MAT of CPGS in the combination group showed greater degrees of decline. The *T*_max⁡_ and CL/F of CPGS in the combination group were slightly higher than those in the clopidogrel-only group, although the difference was not statistically significant. From the results above, we concluded that an apparent herb-drug pharmacokinetic interaction existed between clopidogrel and FDDP, and the combination of clopidogrel and FDDP noticeably altered the absorption, distribution, and disposition of the CPGS.

## 4. Conclusions

FDDP, a common TCM used to activate blood circulation to promote blood stasis, has been clinically used for the treatment of CAD alone or in combination with clopidogrel. However, research is required to ensure its safe clinical use. Because up to 85% of the absorbed parent clopidogrel can be transformed by CES1 into an inactive CPGS, the partial inhibition of the activity of CES1 would result in the decline of CPGS production. It is therefore urgent to investigate the rationality of the synergistic interactions between the two drugs on the metabolism target and pharmacokinetics.

One hundred twenty-seven bioactive components were identified in FDDP, and the max, mean, median, min, mode, variance, and standard deviation values of some key molecular descriptors including AlogP, ROF, HBA, HBD, MR, MW, PHOA, PSA, Polarity, and RB were calculated to evaluate the drug- (lead-) like and PK-PD-like properties of FDDP. Based on the glide energy, docking score, and H-bond interactions with the CES1 target's amino acid residues, 20 compounds in FDDP have potential interactions with the CES1 target. (The original bound ligand THA's value of the CES1 receptor was set as the cutoff.) These results indicated that multiple CES1 inhibitors contained in FDDP and the activity of CES1 activity may be partially inhibited by FDDP through molecular simulation investigations, which may result in the decline of the CPGS concentrations of the CES1 induction pathway.

A reliable HPLC method was developed for quantization of CPGS in rat plasma with a linear range of 0.31–20.0 *μ*g/mL (*r* ^2^ > 0.999). The LLOQ was determined to be 0.31 *μ*g/mL. The intraday and interday precisions were all less than 10.0%, whilst the accuracy values were all within 97.04%–106.22%. The SD rats were treated with oral clopidogrel alone at a dosage of 30 mg/kg or the combination of clopidogrel and FDDP at a dosage of 30 mg/kg and 324 mg/kg, respectively, for 21 days. The CPGS showed a two-compartment model pharmacokinetic profile. The concentration-time course of CPGS was slightly changed by FDDP. Also, the peak plasma concentration and area under the curve of CPGS were decreased by FDDP. The present result demonstrated the synergistic effects between clopidogrel and FDDP by modulating metabolism target and pharmacokinetics. The pharmacokinetics result is relevant to the metabolism enzyme simulation test. The coadministration of clopidogrel with FDDP can cause significant pharmacokinetic herb-drug interactions. The above result demonstrated the synergistic effects of clopidogrel and FDDP by modulating the metabolism target and pharmacokinetics.

## Figures and Tables

**Table 1:** Gradient program used for the separation of CPGS analytes in detection.

	Time (min)				
	0	2	6	8	12
Acetonitrile (%)	14	14	40	14	14
0.2% phosphoric acid (%)	86	86	60	86	86

**Table 2:** The max, mean, median, min, mode, and standard deviation (SD) values of the key descriptors for the one hundred twenty-seven compounds found in the FDDP.

	Minimum	Maximum	Mean	Median	Mode	Standard deviation
AlogP	−4.74	10.86	3.70	3.80	−0.62	2.74
Estate	21.50	273.99	63.31	50.25	24.83	51.35
ROF	0	3	0.74	0	0	0.94
HBA	0.00	27.00	4.08	3.00	0.00	5.36
HBD	0.00	17.00	2.03	0.00	0.00	3.60
MR	18.98	288.70	97.88	82.97	67.26	52.32
MW	116.07	827.38	352.39	284.48	204.35	222.60
PHOA	0	100	79.47	100	100	34.75
PSA	0.00	436.21	73.96	43.37	0.00	90.98
Polar	8.06	117.25	40.79	35.94	26.37	21.18
RB	0.00	20.00	4.72	1.00	0.00	5.62

AlogP: calculate atomic logP; Estate: calculate electrotopological states; HBA: number of hydrogen bond acceptors; HBD: number of hydrogen bond donors; MW: calculate molecular weight; MR: calculate molar refractivity; PHOA: percent human oral absorption; Polarity: calculate Miller polarisability; PSA: calculate polar surface area;RB: calculate the number of rotatable bonds; ROF: rule of five.

**Table 3:** The first 20 molecules that may potentially inhibit human liver carboxylesterase 1.

Title	G-ecoul	G-evdw	G-rewards	G-lipo	G-score	D-score
Tanshinone II A	−0.226	−30.403	−2.828	−4.043	−8.425	−8.425
Tanshinone II B	−1.996	−23.985	−2.596	−4.302	−8.362	−8.362
Delta 1-dehydrotanshinone	−0.195	−26.734	−2.770	−4.178	−8.315	−8.315
Cryptoacetalide	−0.889	−30.915	−2.591	−4.016	−8.286	−8.286
Tanshindiol A	−9.730	−17.620	−2.437	−3.678	−8.265	−8.265
Hydroxytanshinone II A	−5.205	−19.674	−2.381	−4.118	−8.264	−8.264
*zeta*-Cadinene	−7.670	−15.187	−2.250	−3.689	−8.169	−8.169
Nortanshinone	−0.701	−31.512	−3.257	−3.227	−8.165	−8.165
Salvinone	−0.807	−26.017	−2.179	−4.488	−8.088	−8.088
Epidanshenspiroketallactone	−1.332	−26.315	−2.255	−4.273	−8.069	−8.069
Isotanshinone II	−0.421	−25.454	−2.642	−4.014	−7.992	−7.992
Tanshinone I	0.083	−28.932	−2.664	−3.877	−7.975	−7.975
Danshexinkum C	−0.538	−34.893	−2.533	−3.607	−7.966	−7.966
Tanshinlactone	−0.707	−25.399	−2.084	−4.452	−7.912	−7.912
Tanshindiol B	−3.723	−20.496	−2.455	−3.869	−7.907	−7.907
Miltirone	−0.379	−26.509	−2.599	−3.805	−7.786	−7.786
3*α*-Hydroxytanshinone II A	−1.566	−20.607	−2.409	−4.084	−7.758	−7.758
Dihydrotanshinlactone	0.284	−28.718	−1.939	−4.303	−7.636	−7.636
Epidanshenspiroketallactone II	−2.382	−25.089	−2.226	−3.710	−7.633	−7.633
Dihydroisotanshinone I	−1.448	−24.575	−2.432	−3.622	−7.631	−7.631

G-ecoul: Coulomb energy; G-evdw: Van der Waals energy; G-rewards: various rewards or penalty terms; G-lipo: lipophilic contact plus phobic attractive term in the glide score; G-score: glide score; D-score: docking score, including all additional terms.

**Table 4:** Recovery of CPGS in spiked plasma.

Concentration (*μ*g*·*mL^−1^)	Extraction recoveries (%)
0.5	105.12 ± 4.81
5.0	95.31 ± 4.65
20.0	97.50 ± 5.32

**Table 5:** Precision and accuracy of CPGS in spiked rat plasma.

Concentration (*μ*g*·*mL^−1^)	Intraday			Interday		
	Found (*μ*g*·*mL^−1^)	Accuracy (%)	RSD (%)	Found (*μ*g*·*mL^−1^)	Accuracy (%)	RSD (%)
0.5	0.49	98.72	4.39	0.48	97.04	4.78
5.0	5.21	104.28	3.03	5.31	106.22	3.40
20.0	20.23	101.15	1.10	20.13	100.69	1.25

**Table 6:** Pharmacokinetic parameters of the carboxylic acid metabolite of clopidogrel after intragastric administration of clopidogrel alone or coadministered with Fufang Danshen to rats at doses of 324 mg/kgand 30 mg/kg, respectively.

Parameter	Administered alone	Coadministration
*T*_max⁡_ (h)	1.208 ± 0.332	1.583 ± 0.376
*C*_max⁡_ (mg/L)	15. one hundred twenty-seven ± 3.809	10.146 ± 4.848
AUC (0–tn) (mg/L∗h)	90.529 ± 18.557	70.314 ± 35.213
AUC (0–∞) (mg/L∗h)	104.965 ± 24.367	76.324 ± 39.971
MRT (0–tn) (h)	7.453 ± 0.747	6.871 ± 0.741
MRT (0–∞) (h)	11.359 ± 2.022	8.854 ± 1.873^*^
MAT (h)	13.336 ± 2.566	8.198 ± 3.556^*^
Ka	6.807 ± 6.698	2.519 ± 3.073
*V*/*F* (L/kg)	5.079 ± 2.153	9.987 ± 9.820
*T*1/2*β* (h)	14.788 ± 14.311	19.495 ± 18.931
CL/*F* (L/h/kg)	0.309 ± 0.125	0.455 ± 0.184

*C*_max⁡_ (mg/L): maximum plasma concentration; *T*_max⁡_ (h): time to reach maximum concentration; MRT: mean residence time; MAT: mean absorption time; *V*/*F*: apparent distribution volume; CL/*F*: apparent clearance; AUC: the area under the concentration time curve; (0–∞): from time zero to infinity; (0–tn): from time zero to last sampling time; ^*^ *P* < 0.05 when compared with the related parameters of analyte in the clopidogrel-only group.
